# Inhibition of p38 mitogen-activated protein kinase alters microRNA expression and reverses epithelial-to-mesenchymal transition

**DOI:** 10.3892/ijo.2013.1814

**Published:** 2013-02-08

**Authors:** JAMES W. ANTOON, ASHLEY M. NITZCHKE, ELIZABETH C. MARTIN, LYNDSAY V. RHODES, SEUNGYOON NAM, SCOTT WADSWORTH, VIRGILO A. SALVO, STEVEN ELLIOTT, BRIDGETTE COLLINS-BUROW, KENNETH P. NEPHEW, MATTHEW E. BUROW

**Affiliations:** 1 Department of Medicine, Section of Hematology and Medical Oncology, Tulane University School of Medicine, New Orleans, LA 70112;; 2 Department of Cellular and Integrative Physiology, Indiana University School of Medicine, Bloomington, IN 47405;; 3 Johnson & Johnson Pharmaceutical Research & Development, Raritan, NJ 08869, USA

**Keywords:** p38 mitogen-activated protein kinase, epithelial-tomesenchymal transition, breast cancer, drug discovery

## Abstract

Acquired chemoresistance and epithelial-to-mesenchymal transition (EMT) are hallmarks of cancer progression and of increasing clinical relevance. We investigated the role of miRNA and p38 mitogen-activated protein kinase (MAPK) signaling in the progression of breast cancer to a drug-resistant and mesenchymal phenotype. We demonstrate that acquired death receptor resistance results in increased hormone-independent tumorigenesis compared to hormone-sensitive parental cells. Utilizing global miRNA gene expression profiling, we identified miRNA alterations associated with the development of death receptor resistance and EMT progression. We further investigated the role of p38 MAPK in this process, showing dose-dependent inactivation of p38 by its inhibitor RWJ67657 and decreased downstream ATF and NF-κB signaling. Pharmacological inhibition of p38 also decreased chemoresistant cancer tumor growth in xenograft animal models. Interestingly, inhibition of p38 partially reversed the EMT changes found in this cell system, as illustrated by decreased gene expression of the EMT markers Twist, Snail, Slug and ZEB and protein and mRNA levels of Twist, a known EMT promoter, concomitant with decreased N-cadherin protein. RWJ67657 treatment also altered the expression of several miRNAs known to promote therapeutic resistance, including miR-200, miR-303, miR-302, miR-199 and miR-328. Taken together, our results demonstrate the roles of multiple microRNAs and p38 signaling in the progression of cancer and demonstrate the therapeutic potential of targeting the p38 MAPK pathway for reversing EMT in an advanced tumor phenotype.

## Introduction

Acquired chemoresistance is a major cause of clinical treatment failure and cancer mortality. Chemotherapeutic agents and death receptor ligands (e.g., tumor necrosis factor, TNF and Fas), induce cell death through activation of specific cytotoxic pathways (1–3). Chemotherapeutic-induced cell death involves activation of death receptor cascades resulting in programmed cell death (4–6). Resistance to clinical chemotherapeutics often involves alterations in the death receptor signaling cascade to promote survival, rather than apoptosis, in response to these agents (7,8). Acquired chemoresistance is often accompanied by an increase in cellular growth pathways and enhanced metastatic potential (9). In the case of breast cancer, resistance often correlates with the loss of estrogen receptor expression and activation of ER (ERα isoform unless otherwise specified) independent growth pathways, such as PI3K/Akt and mitogen-activated protein kinases signaling cascades, as well as the induction of epithelial-to-mesenchymal transition (EMT) (10–13).

Mitogen-activated protein kinase pathway (MAPK) consists of Erk1/2, JNK, p38 and Erk5/BMK family members (14). These kinases are activated in response to upstream MAPK-kinases (MEKs), which phosphorylate MAPKs in response to specific cellular signals (15–17). Activation of MAPKs, and p38 in particular, results in phosphorylation of selective downstream targets leading to regulation of genes involved in diverse cellular events and have more recently been intensely studied for their critical roles in cancer promotion and progression. By regulating gene expression in response to specific extracellular stimuli, including cytokines (TNF and TRAIL), and known cytotoxic drugs (18), this signaling kinase also promotes transcription of genes involved in invasion, metastasis and survival (19–22). Moreover, p38 activates known downstream transcriptional regulators, including the NF-κB and ATF2 (21,23,24). NF-κB, which is known to promote survival and metastasis, can be directly activated by p38 or phosphorylated by Akt in a p38-dependent manner (21,25). Our laboratory has previously shown that increased basal and activated p38 MAPK signaling is critical to death receptor resistance (26–29). Given the interaction between the p38 MAPK pathway and TNF-NF-κB signaling, the role of p38 in acquired apoptosis resistance is of both biological and therapeutic interest. However, the role of p38 in the development of a mesenchymal phenotypic remains unclear.

MicroRNAs are now well recognized as important regulators of mRNA processing. However, their pathological role in disease processes has only recently come under investigation, with a growing number of studies reporting miRNAs to play roles in tumorigenesis, metastasis and chemoresistance (30–32), and the regulation of EMT (33). Consequently, in this study, we investigated global miRNA expression changes involved in both death receptor and chemotherapy resistance, utilizing mesenchymal MCF-7TN-R cells that are completely resistant to TNF and chemotherapeutics (34–39). Further, we examined the role of p38 MAPK in EMT progression using the p38 inhibitor RWJ67657 (40–42). Given the need for chemoresistant cancer treatment strategies, the development of novel therapeutic targets is of increasing importance and here we demonstrate the therapeutic potential of targeting the p38 MAPK pathway in the treatment of invasive, chemoresistant breast cancer.

## Materials and methods

### Cell generation and culture

MCF-7 cells and MCF-7TN-R were cultured as previously described (43,44). We previously gene rated MCF-7TN-R cells by exposing MCF-7 cells to increasing concentrations of TNFα until resistance was established (35). The culture flasks were maintained in a tissue culture incubator in a humidified atmosphere of 5% CO_2_ and 95% air at 37°C.

### miRNA microarray analysis

MicroRNA analysis was performed as previously described (45). Briefly, MCF-7TN-R cells were plated at a density of 2×10^6^ cells in 25 cm^2^ flasks in normal culture media (DMEM media supplemented with 5% FBS, 1% penicillin/streptomycin, 1% essential amino acids, 1% non-essential amino acids and 1% sodium pyruvate), and then allowed to adhere overnight at 37°C, 5% CO_2_ and air. The following day the media were changed to phenol red-free media (supplemented as above) and 5% CS-FBS. Cells were harvested in PBS, collected by centrifugation, and total-RNA, including small RNA, extracted using the miRNeasy kit (Qiagen, Valencia, CA) according to manufacturer’s protocol, although miRNA enrichment was not performed. Quantity and quality of RNA was determined by absorbance (260, 280 nm), and 5 *μ*g total-RNA was used for microarray analysis. Microarray was used to determine miRNA expression, using three biological replicates for each cell line. Low intensity probes (signal <100 in more than half samples) were excluded from the analysis. Raw data was log-transformed and normalized by IQR (spell out). Clustering of miRNA expression data was performed using cluster analysis (20), with filtering to remove inconsistencies between replicates. For clustering, we first log-transformed the data and median-centered the array and genes, followed by average linkage clustering, with clustering results visualized by TreeView (http://rana.lbl.gov/EisenSoftware.htm). Significant array changes are shown in Table I.

### Real-time RT-PCR

Real-time RT-PCR was performed similarly to previously reported studies (38,46). In brief, total cellular RNA was extracted using RNeasy mini columns (Qiagen), following the manufacturer’s instructions. Reverse transcription (RT) was performed using SuperScript First-Strand (Invitrogen). Gene transcript levels were determined using the iQ5 real-time quantitative PCR detection system (BioRad Inc., Hercules, CA). Quantification and relative gene expression were calculated with internal controls using the 2^−ΔΔCt^ method (47), with the ratio between these values used to determine relative gene expression levels. Primer sequences are available upon request.

### Western blot analysis

Protein expression analysis was performed similarly to previously published methods (39,48). MCF-7TN-R cells were plated at 5×10^5^ cells in T-25 culture flasks and treated as indicated. Cells were harvested, total protein transferred to membranes, and membranes blocked with PBS-Tween (0.05%)-5% low-fat dry milk solution at room temperature for 1 h. The membranes were subsequently probed with the phosphorylated p38 (1:1,000; Cell Signaling, Boston, MA) or phosphorylated ATF-2 (1:1,000; Cell Signaling). Following incubation, blots were washed in PBS-Tween (0.05%) solution and incubated with goat-anti-rabbit antibodies (1:10,000 dilution) for 2 h at room temperature. Following four washes, immunoreactive proteins were detected using the ECL chemiluminescence system (Amersham/GE Healthcare, Pittsburgh, PA) and recorded by fluorography on Hyperfilm (Amersham/GE Healthcare), according to manufacturer’s instructions.

### Reporter gene assays

Reporter gene assays were performed as previously described (49,50). MCF-7TN-R cells were plated at 50,000 cells per 24-well plate and allowed to attach overnight. The next morning, cells were transfected with 50 ng of Gal-4-luciferase (Stratagene, La Jolla, CA) in combination with 25 ng of Gal-4-CHOP (Stratagene) using Effectene reagent for 5 h, according to the manufacturer’s protocol. For NF-κB-luciferase experiments, cells were transfected with 10 ng of pFC-NF-κB-luciferase plasmid (Stratagene). For all luci ferase assays, cells were then incubated for 18–24 h in DMEM with 10% FBS in the presence of vehicle, RWJ or TNF as previously described (22). Following treatment, the media were removed and 150 *μ*l of 1X lysis buffer (Promega, Madison, WI) was added to each well for 1 h on a rocker at room temperature. Luciferase activity for 25 *μ*l of cell extracts was determined using Promega Luciferase System (Promega) in a Berthold AutoLumat Plus luminometer (Wildbad, Germany). For each experiment, changes in luciferase activity were determined as relative light units (RLU) and represented as percent transcriptional activation normalized to vehicle control samples.

### Clonogenic survival assay

As previously described (34,39). MCF-7TN-R cells were plated in 6-well plates at 1×10^3^ cells per well in complete media (10% DMEM). Twenty-four hours later the cells were pre-treated with vehicle (DMSO) or the MAPK inhibitor RWJ67657 (Johnson and Johnson Pharmaceutical Research & Development, L.L.C., Raritan, NJ), as indicated. The cells were then monitored microscopically for colony growth and 7–14 days later were fixed by adding glutaraldehyde (2.5% final concentration) directly to the well. Following fixation for 30 min, the plates were washed and stained with a 0.4% solution of crystal violet in 20% methanol for 30 min, washed and allowed to dry. Colonies greater than 50 were counted and data normalized as percent clonogenic survival from vehicle control cell. For the transfection-based clonogenic survival assay, MCF-7TN-R cells were plated as above. The following day the cells were transfected using the Effectene method (Qiagen) with increasing concentrations of a DI-P38α or β expression vector (0 to 300 ng/well) with total DNA concentrations increasing to 300 ng/well with empty vector. Twenty-four hours later the cells were treated as indicated in the figure legends. The cells were then monitored microscopically for colony growth and harvested and quantitated as above for clonogenic survival.

### Animal studies

Xenograft tumor studies were conducted as previously described (42,51). Immune-compromised female ovariectomized mice (29–32 days old) were obtained from Charles River Laboratories (Wilmington, MA). The animals were allowed a period of adaptation in a sterile and pathogen-free environment with food and water *ad libitum*. MCF-7 and MCF-7TN-R cells were harvested in the exponential growth phase and viable cells mixed with Matrigel Reduced Factors (BD Biosciences, San Jose, CA). Injections (5×10^6^ cells/injection) were made bilaterally into the mammary fat pad. All the procedures in animals were carried out under anesthesia using a mix of isofluorane and oxygen delivered by mask. Tumor sizes were measured twice weekly using a digital caliper, with tumor volumes calculated using the following formula: 4/3πLM^2^, where L is the larger radius, and M is the smaller radius. At necropsy on day 21, animals were euthanized by cervical dislocation after CO_2_ exposure. Tumors were removed and either frozen in liquid nitrogen or fixed in 10% formalin for further analysis. All animal procedures were conducted in compliance with State and Federal laws, standards of the US Department of Health and Human Services, and guidelines established by the Tulane University Animal Care and Use Committee. The facilities and laboratory animal programs of Tulane University are accredited by the Association for the Assessment and Accreditation of Laboratory Animal Care.

### Statistical analysis

Studies involving more than 2 groups were analyzed by one-way ANOVA with Tukey’s post-test using the Graph Pad Prism V.4 software program (Horsham, PA). All others were subjected to unpaired Student’s t-test. A value of p<0.05 was considered statistically significant (52–54).

## Results

### Death receptor resistance promotes hormone independent tumorigenesis

**Figure 1:**
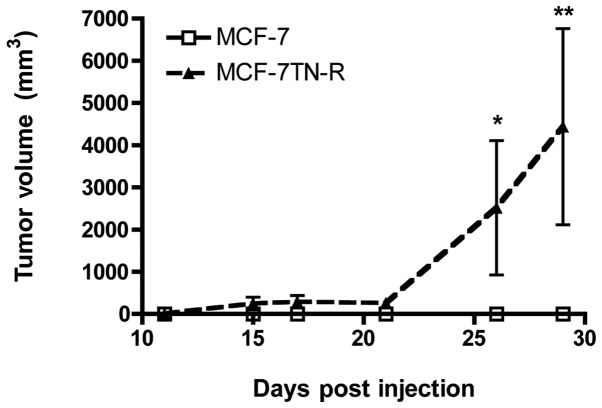
Death receptor resistance promotes hormone independent tumori-genesis. MCF-7 and MCF-7TN-R cells were injected in the mammary fat pads of female ovariectomized mice and measured for tumor formation (^*^p<0.05).

Death receptor-resistant and ER(−) MCF-7TN-R cells exhibit reduced apoptosis, increased survival and multi-drug chemoresistance, as compared to their parental ER(+) MCF-7 cells (35,55). Given the clinical association of chemo-resistance with hormone independence, we investigated the ability of MCF-7TN-R cells to form tumors in the absence of estrogen (56–58). MCF-7TN-R and MCF-7 cells were injected subcutaneously into the flanks of NOD-SCID mice and measured for tumor growth. As seen in Fig. 1, MCF-7TN-R cells showed increased tumor formation and tumor growth compared to MCF-7 cells. The lack of MCF-7 tumor formation in the absence of estrogen correlates with previously published studies (59). These results demonstrate that TNF resistance increased hormone-independent tumorigenesis *in vivo*.

### Global microRNA profiling associated with death receptor resistance

**Figure 2:**
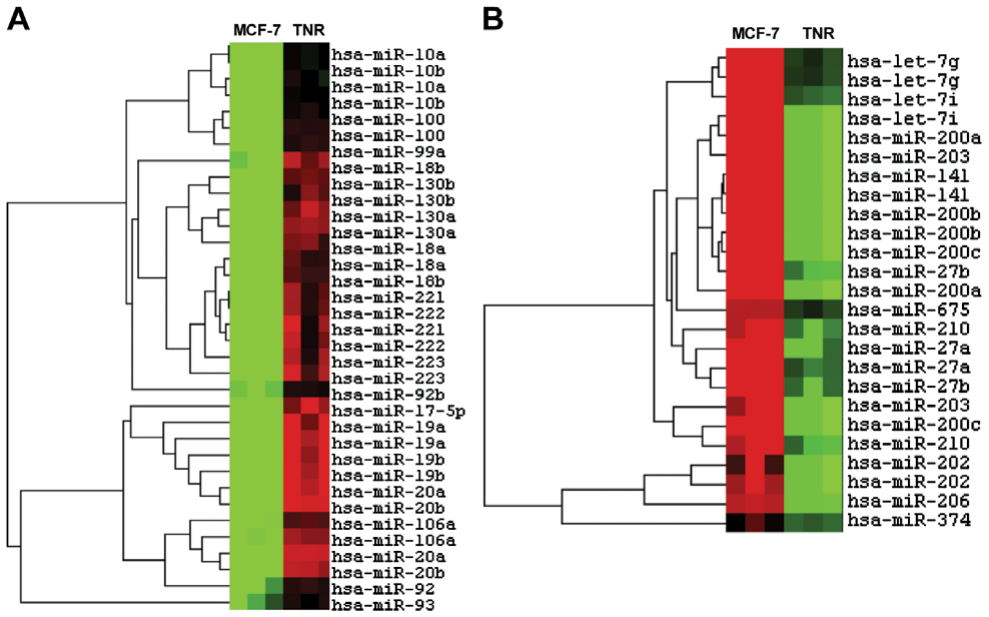
Clustering analysis of microRNA expression profiles of MCF-7 and MCF-7TN-R cells. MCF-7 and MCF-7TN-R have distinctive microarray expression patterns, with samples of same cell lines clustered together. (A) Upregulated miRNA relative to MCF-7. (B) Downregulated miRNA relative to MCF-7. Trees on the left are gene clusters. Red color indicates upregulation and green color indicates downregulation.

We next investigated the mechanism of the increased tumorigenesis seen in MCF-7TN-R cells. miRNAs are small RNAs that target mRNAs for degradation or prevent translation. Several miRNAs have been found to be involved in breast cancer tumorigenesis, including miR-221/222 and the miR-200 family (60). Heatmap analysis demonstrated differential microRNA expression between MCF-7 and its daughter MCF-7TN-R cells (Fig. 2). Specific microRNA expression changes (Table I) demonstrated downregulation of several miRNA associated with tumor suppression, including miR-16, miR-424, miR-15 and miR-19. There was also decreased expression of miR-200, which is strongly associated with breast cancer tumorigenesis. miR-182, miR-183, miR-23 and miR-27, which all target the tumorigenic MEF2C transcription factor, were also downregulated. Furthermore, we noted downregulation of miR-203, a recently identified tumor suppressor (61). These data demonstrate miRNA expression profiles indicative of enhanced tumorigenesis and a more aggressive phenotype in death receptor-resistant cells vs. their parental MCF-7 cells.

We recently demonstrated that chemoresistant MCF-7TN-R cells exhibit increased EMT, resulting in a mesenchymal phenotype (36). As miRNA dysregulation contributes to human breast cancer development (60,62,63), we hypothesized that changes in miRNA expression may also contribute to EMT progression. Microarray results were analyzed for changes in expression of levels of miRNAs whose targets are associated with EMT. Results showed downregulation of several miRNAs known to regulate EMT, including miR-203 and miR-26, which target Slug and Lef, respectively, and miR-200, which targets ETS1, ZEB1 and ZEB2. The metastasis-promoting miR-10b, by contrast (associated with the EMT transcription factor Twist), was markedly upregulated (30).

Our laboratory has previously shown increased basal and stimulated p38 MAPK signaling in MCF-7TN-R cells. As p38 MAPK is known to promote breast cancer hormone independence and EMT progression (24), we hypothesized that changes in miRNA expression may increase p38 signaling in these resistant cells. Therefore, we next analyzed our microarray findings for alterations in miRNA associated with p38 MAPK (64–67). Results revealed increased expression of miRNA associated with increase p38 activity, including miR-34 (a p53 target), miR-17, miR-9, miR-199 (a tumor suppressor that targets c-Met), miR-125 (previously found to be a tumor suppressor in breast cancer), which associate with increased p38 MAPK activity (68–72). Furthermore, compared to parental MCF-7 cells, miR-21 was markedly decreased, in inverse correlation with p38 signaling (73). Together, these results suggest that the increased p38 MAPK activity in MCF-7TN-R cells may in part result from differential microRNA expression.

### RWJ67657 inhibits p38 activation and blocks downstream MAPK signaling

**Figure 3:**
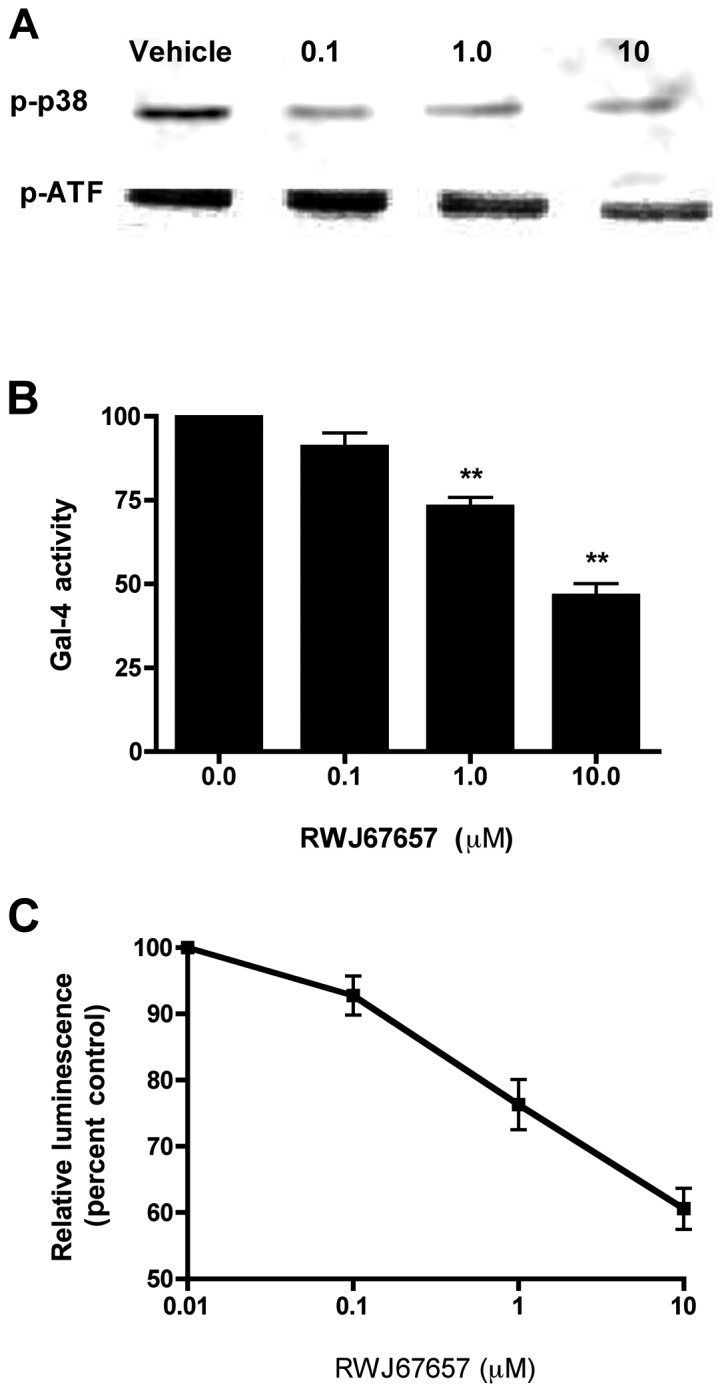
RWJ67657 inhibits p38 signaling. (A) MCF-7TN-R cells were treated with increasing concentrations of RWJ67657 (0–10 *μ*M) for 6 h and harvested for western blot analysis with anti-phospho-p38 or anti-phospho-ATF2 antibodies. (B) MCF-7TN-R cells were transfected with Gal4-CHOP and Gal4-luciferase for 6 h followed by treatment with increasing concentrations of RWJ67657 (0–10 *μ*M). The following day cells were harvested for luciferase assay. Data points and error bars represent the mean ± SEM of three independent experiments in triplicate (^**^p<0.01). (C) MCF-7TN-R cells were transfected with NF-κB-luciferase for 6 h and pre-treated with increasing concentrations of RWJ67657 (0–10 *μ*M) followed by treatment with or without TNF (1.0 ng/ml) overnight. The following day cells were harvested for luciferase activity. Data points and error bars represent the mean ± SEM of three independent experiments in triplicate.

The microRNA profile above correlates with increased MAPK signaling previously observed in these cells (27). Given the role of p38 signaling in breast cancer promotion and progression, we further investigated the therapeutic potential of targeting this pathway by determining whether p38 pharmacological inhibition could reverse the neoplastic changes found in these cells. To that end, we used the p38 inhibitor RWJ67657 for investigating suppression of p38 activity. We first determined RWJ67657 blockage of p38 signaling in chemoresistant breast cancer cells. MCF-7TN-R cells, exposed to increasing concentrations of RWJ67657, were analyzed for phosphorylated p38 by western blot analysis (Fig. 3A). In comparison to vehicle treated cells, treatment with RWJ67657 resulted in a dose-dependent decrease phosphorylation of p38 MAPK. Exposure to RWJ67657 had no effect on total levels of p38 (data not shown), suggesting that RWJ67657 blocks activation, but not basal expression, of p38 and ATF, a downstream effector of p38 signaling.

We next determined whether inhibition of p38 by RWJ67657 might also decrease pathway-downstream transcriptional activators. MCF-7TN-R cells were treated with RWJ67657 and activation of the p38 pathway was determined by measurement of Gal-CHOP transactivation (Fig. 3B). RWJ67657 decreased p38 transcriptional activity in a dose-dependent manner, suggesting functional blockage of p38 MAPK signal transduction. Additionally, we also analyzed the ability of RWJ67657 to block that NF-κB signaling, a known downstream target of p38 (21,24,27,74). As shown in Fig. 3C, dose-dependent RWJ67657 treatment decreased NF-κB transcriptional activity. Taken together, these findings provide proof-of-principle that RWJ67657 blocks p38 MAPK signaling found in MCF-7TN-R cells.

### Inhibition of p38 suppresses clonogenic survival and in vivo tumor growth

**Figure 4:**
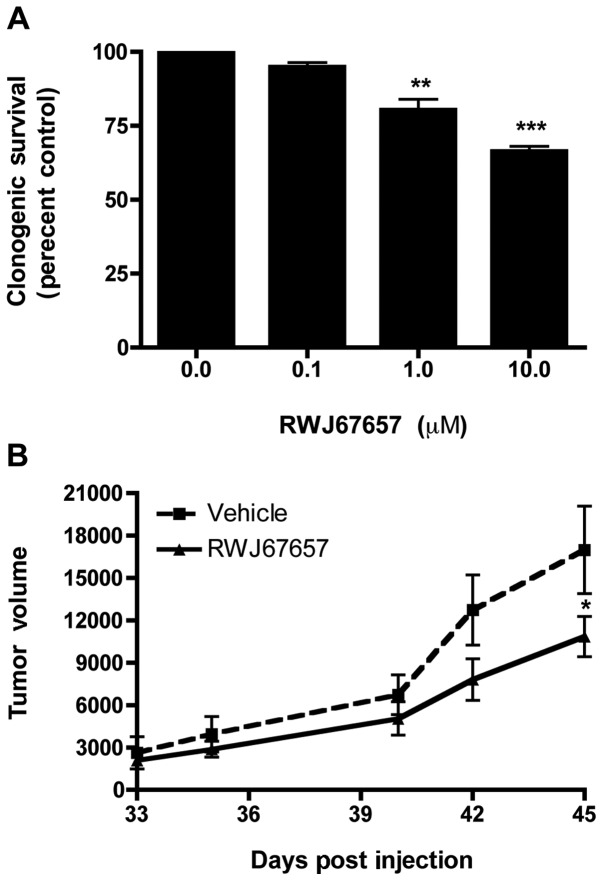
Inhibition of p38 blocks clonogenic survival and suppresses tumor growth. (A) MCF-7TN-R cells were plated for clonogenic survival assay, treated with increasing concentrations of RWJ67657 (0–10 *μ*M) and allowed to grow until visible colonies appeared. Cells were then harvested, stained and counted for clonogenic survival. Data points and error bars represent the mean ± SEM of three independent experiments in triplicate (^**^p<0.01, ^***^p<0.001). (B) MCF-7TN-R cells (5×10^5^) were injected s.c. into immune-compromised mice. The mice treated with vehicle control or RWJ67657 (60 mg/kg) for 9 days. Tumor volume was monitored biweekly after palpable tumor formation (^*^p<0.05).

Our laboratory has previously demonstrated increased colony formation and long-term survival of MCF-7TN-R cells, as compared to their parental MCF-7 (35). Therefore, to determine whether p38 MAPK inhibition could suppress *in vitro* survival of MCF-7TN-R cells, cells treated with increasing concentrations of RWJ67657 were analyzed for long-term colony formation. As shown in Fig. 4A, treatment with RWJ67657 inhibited clonogenic survival in a dose-dependent manner. We next sought to validate our clonogenic survival results using a xenograft animal tumor model. MCF-7TN-R cells were implanted into the mammary fat pad of female immune-compromised, treated with either vehicle control or RWJ67657, and monitored for tumor formation. Fig. 4B illustrates that treatment with RWJ67657 resulted in a statistically significant decrease in tumor volume compared to vehicle-treated animals in our chemoresistant xenograft model. These results suggest that targeting p38 may be therapeutically relevant in the treatment of death ligand-resistant breast cancer.

### p38 inhibition reverses epithelial-to-mesenchymal transition

**Figure 5:**
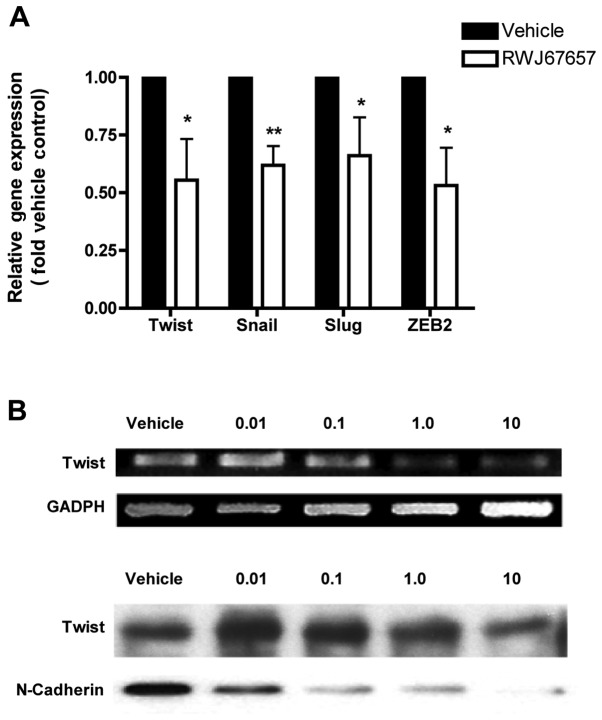
Inhibition of p38 reverses epithelial-to-mesenchymal transition. (A) mRNA gene expression of the EMT markers Twist, Snail, Slug and ZEB2 were quantified using qPCR in MCF-7TN-R cells following treatment with RWJ67657 (10 *μ*M) for 24 h. Mean values ± SEM of three independent experiments in duplicate are reported (^**^p<0.01, ^*^p<0.05). (B) RT-PCR analysis of Twist or GADPH (control) expression and western blot analysis of Twist and N-cadherin expression following treatment with indicated concentrations of RWJ67657 (0–10 *μ*M) for 24 h. Blots shown are representative of three independent experiments.

Our above microRNA findings suggest an increased EMT phenotype in MCF-7TN-R cells, consistent with previously published findings (36). Given the ability of RWJ67657 to inhibit MCF-7TN-R survival and tumor growth, we next determined whether inhibition of p38 could reverse the EMT changes found in this chemoresistant cell model. MCF-7TN-R cells were treated with RWJ67657 and measured for expression of EMT markers Twist, Snail, Slug and ZEB2, chosen because MCF-7TN-R exhibited increased expression of these EMT genes (36). Treatment with RWJ67657 decreased mRNA expression of all four EMT markers (Fig. 5A), suggesting that EMT in chemoresistant cells is at least partially p38-dependent. We next sought to validate these results using RT-PCR and western blot analysis for Twist. Our microRNA analysis above showed a significant increase in pro-metastasis miR-10b in MCF-7TN-R cells, possible association with Twist expression (30,75,76). Treatment with RWJ67657 dose-dependently decreased Twist mRNA and protein expression (Fig. 5B). A hallmark of EMT in breast cancer cells is expression of the mesenchymal surface protein N-cadherin, which is regulated by Twist (77,78). To confirm possible anti-EMT activity, RWJ67657-treated cells were examined for the EMT marker N-cadherin. Expression of N-cadherin was indeed decreased by dose-dependent RWJ67657. Taken together, these results suggest that pharmacological inhibition of p38 may inhibit the EMT, an advanced cancer phenotype, in chemoresistant MCF-7TN-R cells.

### RWJ67657 alters endogenous microRNA profiling

**Figure 6:**
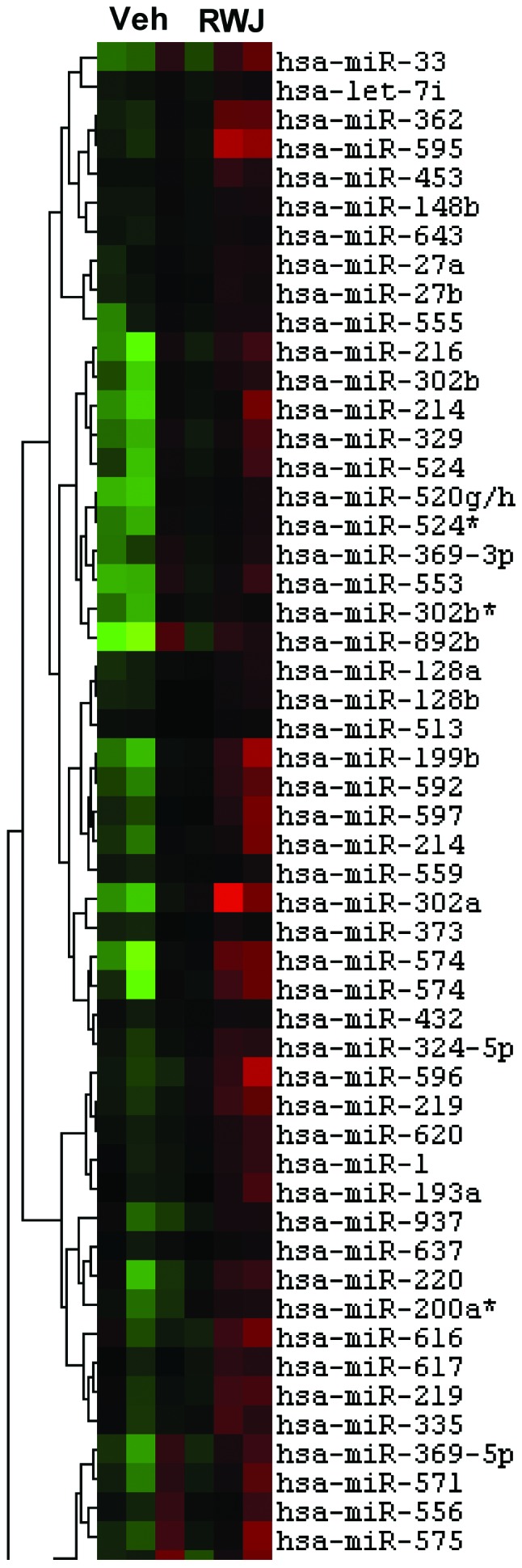
Clustering analysis of microRNA expression profiles of RWJ67657. MCF-7 and MCF-7TN-R have distinctive microarray expression patterns, with samples of same cell lines clustered together. Trees on the left are gene clusters. Red color indicates upregulation and green color indicates downregulation.

Given the differential microRNA expression found in MCF-7TN-R, we next determined whether p38 suppression might alter microRNAs functional in breast cancer cells. Consequently, vehicle- or RWJ67657-treated MCF-7TN-R cells were analyzed for microRNA expression changes. Heatmap analysis demonstrated differential microRNA expression associated with RWJ67657 treatment (Fig. 6). Specific microRNA expression changes are shown in Table II. Treatment with RWJ67657 resulted in changes in expression of several microRNAs known to be involved in cancer chemotherapy and drug resistance. Of note, we found down-regulation of miR-200 and miR-303, which are involved in resistance to doxorubicin (79), as was a known mediator of paclitaxel resistance, miR-302 (80,81). We also found changes in microRNA associated with endocrine therapy resistance, including RWJ67657-altered expression of miR-199, a known effector of fulvestrant resistance, and miR-328, associated with resistance to both fulvestrant and mitoxantrone (81,82). Thus, MCF-7TN-R-misexpressed microRNAs likely contribute to resistance to doxorubicin, paclitaxel and fulvestrant, in addition to their more general alterations associated with the anticancer effects of RWJ67657.

## Discussion

Tumor cell progression to an invasive and chemotherapeutic-resistant phenotype represents a significant obstacle that must be overcome to successfully treat and cure cancer. To investigate mechanisms by which cancer cells progress to a resistant phenotype, we employed a previously established *in vitro* model of acquired resistance (27,35,37,39,83), the cell line MCF-7TN-R, which is resistant to both death receptors and chemotherapeutic drugs that depend on p38 MAPK and NF-κB signaling (27,37,55,84). Further, we investigated whether progression to apoptotic resistance also associated with increased hormone-independent tumor formation and p38 signaling. To delineate possible mechanisms of resistance, we investigated whether alternatively expressed microRNAs allow progression from the chemo-sensitive MCF-7 cell line to the chemoresistant cell line MCF-7TN-R.

Given the increasing evidence that microRNAs play a significant role in cancer, we examined the miRNA profile of parental MCF-7 and resistant MCF-7TN-R cell lines by micro-array analysis. Our results revealed differential expression of several miRNAs involved in the progression to a mesenchymal phenotype, including miR-200 and miR-10b. Specifically, miR-200 is a key regulator of EMT in numerous cancers, promoting an epithelial phenotype by inhibiting several EMT genes, including ZEB1 and ZEB2 (33). Further, we also demonstrated a significant increase in miR-10, which associates with expression of the EMT gene Twist (30,75,76,85). Twist is known to upregulate ER and N-cadherin expression to promote EMT (77,78). The gain of mesenchymal properties via EMT permits cells to detach from one another and migrate through the basement membrane. Our findings here also concur with previously published results demonstrating increased EMT in resistant MCF-7TN-R cells, and further suggest that microRNA changes may promote a mesenchymal phenotype.

Recent studies have linked tumor growth, invasion, and chemoresistance to miRNA alterations (86). Of particular interest, a recent microarray analysis by Chen *et al* comparing a doxorubicin-resistant MCF-7 cell line to the doxorubicin-sensitive MCF-7 parental cell line revealed differential miRNA expression, suggesting that specific miRNAs may modulate chemoresistance in breast cancer (32). The miRNA changes described by Chen *et al* are consistent with the miR-21 and miR-34 changes seen in our MCF-7TN-R cells. Additionally, a recent study by Zhu *et al* showed that molecular inhibition of miR-21 in MDA-MB-231 cells results in suppression of invasion and metastasis (87). We found inhibition of p38-altered expression of miRNAs known to promote both endocrine therapy- and chemo-resistance in these cells, including miR-200, miR-303, miR-302, miR-199 and miR-328 (79–81,82). In light of the recent evidence supporting miRNA changes in aggressive breast cancer EMT, the differential miRNA expression found in our microarray likely contributes to the apoptotic resistance of these cells.

Having previously identified p38-NF-κB-signaling as the mediator of chemoresistance in MCF-7TN-R cells, we hypothesized that this pathway may be responsible for the EMT and increased tumorigenesis seen in the MCF-7TN-R cells (27). To test this hypothesis, we attempted to reverse the changes seen in the MCF-7TN-R cell line by inhibiting this pathway with RWJ67657, a pharmacologic inhibitor of p38. Western blot analysis and reporter gene assays following exposure to the inhibitor revealed decreased p38 activation and downstream signaling, demonstrating that RWJ67657 functionally blocked p38 signaling in our resistant cells. We then evaluated the role of p38 in EMT through utilization of this p38 inhibitor. Treatment with RWJ67657 decreased mRNA expression of the EMT markers Twist, Snail, Slug, and ZEB2. Overall, loss of these markers indicates loss of a mesenchymal markers in MCF-7TN-R cells when treated with RWJ67657. These results agree with recent findings that suggest a role for p38 in EMT, strongly supporting the therapeutic potential of targeting this pathway in EMT breast cancer (88–90). Pharmacological inhibition of p38 was also associated with decreased clonogenic survival in MCF-7TN-R cells *in vitro* and a statistically significant reduction in tumor volume, as compared to vehicle mice, thus indicating that RWJ67657 MAPK inhibition can biologically reverse the aggressive properties seen in MCF-7TN-R cells, in addition to the molecular reversals described above.

Taken together, our findings suggest that p38 MAPK-microRNA signaling is required for the maintenance of an aggressive phenotype in MCF-7TN-R cells, stimulating increased clonogenic survival and tumor growth, along with EMT indicators. Specifically, p38 downstream modulation of EMT-regulator Twist and microRNAs in the p38 pathway appears to play an important role in this process. Further research is warranted to determine the exact mechanism by which these effectors interact. Finally, disruption of the p38 MAPK signaling pathway by the pharmacological inhibitor RWJ67657 reverses this aggressive phenotype, implicating the signaling pathway as a therapeutic target for aggressive breast cancers.

## Figures and Tables

**Table I:** microRNA changes associated with acquired TNF resistance.

Downregulated			Upregulated		
microRNA	Expression (fold MCF-7)	P-value	microRNA	Expression (fold MCF-7)	P-value
hsa-miR-21	−75.47	3.29E-05	hsa-miR-30a-5p	2.02	1.07E-02
hsa-let-7a	−3.37	2.24E-03	hsa-miR-100	5.43	3.84E-04
hsa-let-7a	−3.34	6.35E-03	hsa-miR-100	6.03	2.76E-04
hsa-let-7b	−2.34	2.44E-02	hsa-miR-106a	3.70	5.55E-04
hsa-let-7c	−2.34	3.44E-02	hsa-miR-106a	3.76	5.65E-03
hsa-let-7d	−2.73	2.38E-02	hsa-miR-106b	2.15	2.73E-02
hsa-let-7e	−2.62	1.24E-02	hsa-miR-10a	7.89	2.19E-04
hsa-let-7f	−4.43	4.68E-03	hsa-miR-10a	7.90	1.60E-04
hsa-let-7f	−3.31	2.68E-02	hsa-miR-10b	6.52	2.34E-04
hsa-let-7g	−5.70	2.85E-04	hsa-miR-10b	6.57	2.34E-04
hsa-let-7g	−4.88	1.05E-03	hsa-miR-130a	5.20	7.49E-05
hsa-let-7i	−6.52	1.77E-04	hsa-miR-130a	5.45	3.16E-04
hsa-let-7i	−4.72	6.66E-04	hsa-miR-130b	4.54	5.82E-07
hsa-miR-125a	−2.49	4.22E-02	hsa-miR-130b	4.91	1.28E-04
hsa-miR-125a	−2.46	1.06E-02	hsa-miR-148a	2.06	1.07E-02
hsa-miR-141	−9.28	4.75E-05	hsa-miR-17-5p	4.29	2.56E-04
hsa-miR-141	−8.66	1.28E-04	hsa-miR-17-5p	4.47	5.68E-04
hsa-miR-185	−2.05	1.95E-02	hsa-miR-18a	5.04	7.29E-04
hsa-miR-193b	−2.35	4.30E-04	hsa-miR-18a	5.34	5.25E-04
hsa-miR-193b	−2.32	2.18E-03	hsa-miR-18b	4.11	1.08E-03
hsa-miR-200a	−6.71	6.85E-04	hsa-miR-18b	4.89	4.91E-04
hsa-miR-200a	−6.36	1.77E-05	hsa-miR-19a	4.72	1.21E-03
hsa-miR-200b	−12.57	8.40E-06	hsa-miR-19a	9.06	1.66E-04
hsa-miR-200b	−11.79	1.90E-04	hsa-miR-19b	7.00	7.51E-04
hsa-miR-200c	−14.38	3.23E-05	hsa-miR-19b	7.44	9.85E-04
hsa-miR-200c	−10.24	2.56E-03	hsa-miR-20a	6.05	3.40E-04
hsa-miR-202	−3.74	4.27E-04	hsa-miR-20a	6.87	7.08E-04
hsa-miR-202	−2.94	3.31E-03	hsa-miR-20b	5.21	1.34E-04
hsa-miR-203	−6.11	4.54E-03	hsa-miR-20b	5.87	1.03E-03
hsa-miR-203	−5.47	2.90E-04	hsa-miR-221	12.97	1.76E-04
hsa-miR-206	−3.44	4.89E-08	hsa-miR-221	13.85	6.05E-04
hsa-miR-206	−2.17	3.54E-03	hsa-miR-222	12.61	3.52E-04
hsa-miR-21	−68.35	6.86E-06	hsa-miR-222	12.69	1.70E-04
hsa-miR-210	−3.85	1.39E-03	hsa-miR-223	5.21	1.52E-03
hsa-miR-210	−3.35	4.18E-04	hsa-miR-223	7.97	4.48E-04
hsa-miR-23a	−5.00	2.74E-03	hsa-miR-30a-5p	2.18	6.57E-03
hsa-miR-23a	−4.96	1.17E-02	hsa-miR-30b	2.20	2.92E-02
hsa-miR-23b	−4.83	1.39E-02	hsa-miR-30d	2.07	2.82E-02
hsa-miR-23b	−4.75	3.09E-03	hsa-miR-30d	2.18	1.60E-02
hsa-miR-24	−4.17	3.21E-03	hsa-miR-31	2.02	1.53E-02
hsa-miR-24	−3.86	5.66E-04	hsa-miR-34a	2.07	8.92E-04
hsa-miR-27a	−5.64	1.90E-04	hsa-miR-34a	2.09	3.52E-03
hsa-miR-27a	−5.37	6.21E-05	hsa-miR-9	2.13	9.83E-03
hsa-miR-27b	−6.74	7.10E-05	hsa-miR-92	2.26	1.76E-03
hsa-miR-27b	−6.30	6.34E-04	hsa-miR-92b	2.24	1.52E-03
hsa-miR-29a	−2.02	3.98E-03	hsa-miR-99a	4.59	5.48E-04
hsa-miR-29a	−2.00	2.18E-02			
hsa-miR-29b	−2.59	1.01E-02			
hsa-miR-29b	−2.52	1.11E-02			
hsa-miR-335	−2.10	3.27E-03			
hsa-miR-342	−8.25	2.77E-03			
hsa-miR-342	−7.16	9.04E-03			
hsa-miR-345	−2.27	1.04E-03			
hsa-miR-345	−2.19	1.35E-03			
hsa-miR-425-5p	−2.37	2.02E-02			
hsa-miR-425-5p	−2.00	3.57E-02			
hsa-miR-487b	−2.07	8.62E-04			
hsa-miR-574	−2.37	1.95E-02			
hsa-miR-675	−2.20	4.40E-05			
hsa-miR-7	−3.01	7.11E-03			
hsa-miR-98	−2.92	5.55E-03			

**Table II:** microRNA changes associated with RWJ67657.

microRNA	Expression (fold vehicle control)	P-value
hsa-miR-143	1.62	4.81E-02
hsa-miR-153	−2.38	3.11E-03
hsa-miR-190	−1.76	1.43E-02
hsa-miR-199b	−2.82	4.32E-02
hsa-miR-199b	−2.76	2.86E-02
hsa-miR-200a	−2.91	1.84E-03
hsa-miR-200a	−3.14	4.92E-03
hsa-miR-200a	−1.83	2.01E-02
hsa-miR-200b	−1.94	2.70E-02
hsa-miR-200b	−2.39	2.09E-02
hsa-miR-217	−2.67	4.24E-02
hsa-miR-219	−2.04	2.08E-02
hsa-miR-300	2.07	1.64E-02
hsa-miR-301	−1.94	4.47E-02
hsa-miR-302a	−3.97	3.08E-02
hsa-miR-302a	−2.81	4.32E-02
hsa-miR-302a	−3.05	2.27E-03
hsa-miR-302b	−2.50	3.71E-02
hsa-miR-302c	−2.11	4.79E-02
hsa-miR-324-3p	−2.09	2.58E-02
hsa-miR-324-5p	−1.74	2.69E-02
hsa-miR-325	−2.72	1.00E-02
hsa-miR-328	−2.68	4.36E-02
hsa-miR-33	−2.41	4.06E-02
hsa-miR-335	−1.50	3.12E-02
hsa-miR-362	−3.03	2.78E-03
hsa-miR-363	−2.46	8.92E-03
hsa-miR-488	−3.63	1.45E-02
hsa-miR-492	1.75	1.27E-02
hsa-miR-492	1.83	1.33E-02
hsa-miR-520g/h	−2.79	6.26E-03
hsa-miR-521	−3.05	3.59E-02
hsa-miR-522	−3.52	1.15E-02
hsa-miR-549	−2.25	4.64E-02
hsa-miR-550	−2.91	2.41E-02
hsa-miR-551b	−2.32	3.17E-02
hsa-miR-570	−1.81	2.50E-02
hsa-miR-574	−3.23	4.71E-02
hsa-miR-592	−2.19	4.63E-02
hsa-miR-592	−4.27	1.17E-02
hsa-miR-593	−2.24	4.81E-02
hsa-miR-595	−2.14	4.43E-02
hsa-miR-596	−2.48	2.62E-02
hsa-miR-607	1.68	4.75E-02
hsa-miR-616	−2.48	1.97E-02
hsa-miR-620	−1.58	2.69E-02
hsa-miR-620	−1.58	2.69E-02
hsa-miR-627	−1.59	4.11E-02
hsa-miR-628	−1.53	3.28E-02
hsa-miR-663	1.74	2.88E-02
hsa-miR-871	−3.21	3.39E-02
hsa-miR-871	−5.42	1.45E-02
hsa-miR-886-5p	−1.81	7.97E-04
hsa-miR-888	−1.66	4.44E-02
hsa-miR-891b	1.80	3.41E-02
hsa-miR-922	−2.41	3.90E-02
hsa-miR-922	−5.42	3.14E-03
hsa-miR-923	1.68	9.30E-03
